# Efficacy of Non-Invasive Brain Stimulation for Treating Depression in Patients with Traumatic Brain Injury: A Meta-Analysis and Meta-Regression of Randomized Controlled Trials

**DOI:** 10.3390/jcm12186030

**Published:** 2023-09-18

**Authors:** Chun-Hung Chang, Po-Han Chou, Hao-Yu Chuang, Chi-Yu Yao, Wei-Jen Chen, Hsin-Chi Tsai

**Affiliations:** 1 Institute of Clinical Medical Science, China Medical University, Taichung 406040, Taiwan; chang763@gmail.com; 2 Department of Psychiatry & Brain Disease Research Center, China Medical University Hospital, Taichung 404327, Taiwan; 3 An Nan Hospital, China Medical University, Tainan 709204, Taiwan; ycy945@gmail.com (C.-Y.Y.); yizingch@gmail.com (W.-J.C.); 4 Department of Psychiatry, China Medical University Hsinchu Hospital, China Medical University, Hsinchu 302056, Taiwan; 5 Department of Psychiatry, China Medical University Hospital, China Medical University, Taichung 404327, Taiwan; 6 Department of Neurosurgery, An Nan Hospital, China Medical University, Tainan 709204, Taiwan; greeberg1975@gmail.com; 7 Department of Psychiatry, Tzu-Chi General Hospital, Hualien 970473, Taiwan; 8 Institute of Medical Sciences, Tzu Chi University, Hualien 970473, Taiwan

**Keywords:** non-invasive brain stimulation, depression, transcranial magnetic stimulation, transcranial direct current stimulation

## Abstract

Objective: This meta-analysis aimed to ascertain the efficacy of non-invasive brain stimulation (NIBS)—comprising repetitive transcranial magnetic stimulation (rTMS) and transcranial direct current stimulation (tDCS)—for depression in traumatic brain injury (TBI) patients. Methods: Comprehensive searches were conducted in PubMed, Cochrane Database of Systematic Reviews, and the Cochrane Central Register of Controlled Trials up to 28 January 2023. Random-effects models assessed the treatment effects, and heterogeneity was evaluated through *I*^2^ statistics and funnel plot inspection. Results: From 10 trials (234 participants; 8 rTMS, 2 tDCS), NIBS was found significantly more effective than sham in alleviating depressive symptoms (SMD: 0.588, 95% CI: 0.264–0.912; *p* < 0.001). rTMS, specifically, showed higher efficacy (SMD: 0.707, 95% CI: 0.306–1.108; *p* = 0.001) compared to sham, whereas tDCS outcomes were inconclusive (SMD: 0.271, 95% CI: −0.230 to 0.771; *p* = 0.289). Meta-regression found no correlation with the number of sessions, treatment intensity, or total dose. Notably, while post-treatment effects were significant, they diminished 1–2 months post intervention. Adverse events associated with NIBS were minimal, with no severe outcomes like seizures and suicide reported. Conclusions: rTMS emerged as a potent short-term intervention for depression in TBI patients, while tDCS findings remained equivocal. The long-term efficacy of NIBS is yet to be established, warranting further studies. The low adverse event rate reaffirms NIBS’s potential safety.

## 1. Introduction

Among all prevalent neurological conditions, traumatic brain injury (TBI) has the highest incidence rate worldwide and thus presents a major public health challenge [1]. According to a meta-analysis of 82 studies, TBI has a pooled (all age groups) annual incidence of 295 (95% CI: 274–317) per 100,000 and a pooled incidence rate of 349 (95% CI: 96.2–1266) per 100,000 person-years [2]. In addition to disability and mortality, TBI is also associated with numerous psychiatric sequelae, including depression (9%), generalized anxiety disorder (9%), post-traumatic stress disorder (6%), and agoraphobia (6%) [3]. In a meta-analysis of 16 studies involving 1,146,271 patients with TBI, Chen et al. [4] discovered that TBI was associated with suicidal ideation and suicide attempt prevalence of 19.1% (95% CI: 13.6–24.6%) and 2.1% (95% CI: 1.8–2.4%), respectively. These findings highlight the urgent need for effective treatment strategies for post-TBI depression.

Non-invasive brain stimulation (NIBS), including repetitive transcranial magnetic stimulation (rTMS) and transcranial direct current stimulation (tDCS), may facilitate patient recovery by modulating cortical excitability and increasing dendritic spines and their connections [5,6,7]. These techniques have potential for treating multiple neuropsychiatric disorders, including depression [8,9]. In rTMS, a rapidly changing current is delivered through a coiled, plastic-encased wire positioned above the patient’s scalp. According to Faraday’s law of electromagnetic induction, this setting creates a magnetic field across the skull and subsequently generates an electric current in the targeted brain regions [10,11], resulting in the modulation of cortical excitability [12]. In addition, rTMS delivers trains of pulses in various modalities (e.g., high-frequency [≥5 Hz], low-frequency [≤1 Hz], and theta burst stimulation including intermittent theta burst stimulation and continuous theta burst stimulation) at a consistent intensity over a specified time period [13]. High-frequency pulses and intermittent theta burst stimulation may increase cortical excitability, whereas low-frequency pulses and continuous theta burst stimulation may reduce it [10,12,13]. Depending on its frequency, rTMS modulates cortical excitability, neurotransmitter release, signaling pathways, and gene expression [14,15,16]. By contrast, in tDCS, an electric current (typically 1–2 mA) is delivered through 2 or more electrodes placed on the patient’s scalp [17]. This weak current penetrates the skull and modulates the neural activity of each of the targeted brain regions [18]. This technique may modulate neuronal activity by altering the membrane polarization of neurons [19,20]. Anodal tDCS may increase cortical excitability in the brain region under and around the electrode, whereas cathodal tDCS may reduce it [21].

Although pilot studies have indicated that NIBS has high efficacy for treating depression after brain trauma [22,23,24,25], some studies have indicated that NIBS has no such efficacy whatsoever [26,27]. However, in those studies, factors including frequency, brain target, and total pulses used have not been standardized. Previous meta-analytical studies have focused on the effect of rTMS on post-TBI depression [28,29,30]. Gertler et al. [30] evaluated a study regarding the use of rTMS and tricyclic antidepressants versus that of tricyclic antidepressants alone (standardized mean difference (SMD): −0.84, 95% CI: −1.36 to −0.32, *Z* = 3.19; *p* = 0.001). Beedham et al. [28] evaluated 4 trials involving rTMS and discovered that in those trials, rTMS substantially reduced the severity of depression (SMD: 2.43, 95% CI: 1.24–3.61). Tsai et al. [29] evaluated 7 trials regarding the effects of rTMS on patients with TBI and depression and discovered that rTMS significantly alleviated those patients’ depressive symptoms (SMD: 1.03; *p* = 0.02). By contrast, Annegers and Coan [31] reported that patients with TBI had increased risk of seizure, and Hu et al. [32] indicated that brain stimulation seemed to increase the risk of seizure. However, neither of those reviews analyzed the adverse effects of treatment, especially seizures and suicide. In addition, the correlations of treatment response with factors such as the TBI baseline severity, total number of sessions, total pulses, and treatment intensity remained unclear. Accordingly, pilot studies have investigated the efficacy of tDCS for treating TBI-associated neuropsychological symptoms; however, corresponding results obtained for the effects of tDCS treatment have been conflicting [27,33]. Therefore, we conducted the present meta-analysis to evaluate the efficacy of NIBS, particularly rTMS and tDCS, for treating post-TBI depression. We evaluated adverse effects (especially seizures and suicide). We used subgroup analysis and meta-regression to analyze the moderators including the NIBS type, frequency, brain target, TBI baseline severity, total number of sessions, total pulses, and treatment intensity (defined as % Resting Motor Threshold referring to the minimum amount of magnetic field strength required to elicit a motor-evoked potential in a target muscle).

## 2. Methods

### 2.1. Search Strategy and Study Selection

**Figure 1:**
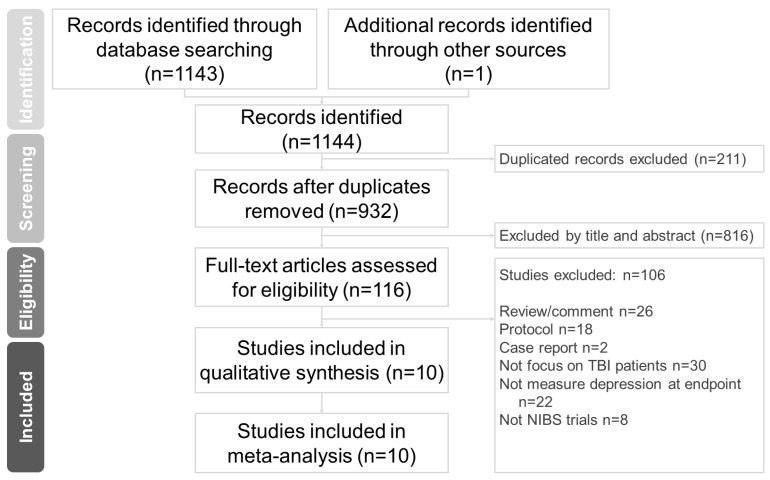
PRISMA flowchart for study selection. Databases: PubMed (*n* = 470), Cochrane Central Register of Controlled Trials (*n* = 655), and Cochrane Database of Systematic Reviews (*n* = 18). Keywords: (traumatic brain injur* OR TBI OR head injur* OR brain injur* OR brain trauma OR concussion OR concussive) AND (tDCS OR transcranial direct current stimulation OR non-invasive stimulation OR transcranial magnetic stimulation OR TMS OR rTMS OR brain stimulation) AND controlled trial. Date: Up to January 2023. Abbreviations: NIBS, non-invasive brain stimulation; PRISMA, Preferred Reporting Items for Systematic Reviews and Meta-Analyses; rTMS, repetitive transcranial magnetic stimulation; TBI, traumatic brain injury; tDCS; transcranial direct current stimulation.

PubMed, the Cochrane Central Register of Controlled Trials, and the Cochrane Database of Systematic Reviews were independently and systematically searched (up to 28 January 2023) by 2 experienced authors (C.-H. Chang and W.-J. Chen) for randomized controlled trials in which NIBS was used to treat TBI. The search terms are listed as follows: (traumatic brain injur* OR TBI OR head injur* OR brain injur* OR brain trauma OR concussion OR concussive) AND (tDCS OR transcranial direct current stimulation OR non-invasive stimulation OR transcranial magnetic stimulation OR TMS OR rTMS OR brain stimulation) AND controlled trial (Table S1) [34]. Relevant original research and review articles were manually searched for pertinent references. This study was conducted in accordance with the Preferred Reporting Items for Systematic Reviews and Meta-Analyses guidelines (Figure 1) [35].

### 2.2. Inclusion and Exclusion Criteria

Studies involving patients with TBI, studies with a randomized trial design (e.g., randomized controlled trials and randomized cross-over trials), and studies involving the use of NIBS as a monotherapy or adjunctive therapy were included in this analysis. Exclusion criteria for the analysis encompassed review articles, commentaries, case reports, and protocols. Additionally, studies not centered on patients with Traumatic Brain Injury (TBI) or not evaluating end-point depression were omitted. Non-NIBS trials were also excluded.

### 2.3. Data Extraction

Data related to the following items were independently extracted by the aforementioned authors from all the retrieved articles: first author’s name, publication year, sample size, sex ratio, mean age, depression measure, baseline mean depression score, NIBS type, brain target, treatment frequency, treatment intensity, total number of sessions, and time since injury (Table 1).

### 2.4. Methodological Quality Appraisal

**Figure 2:**
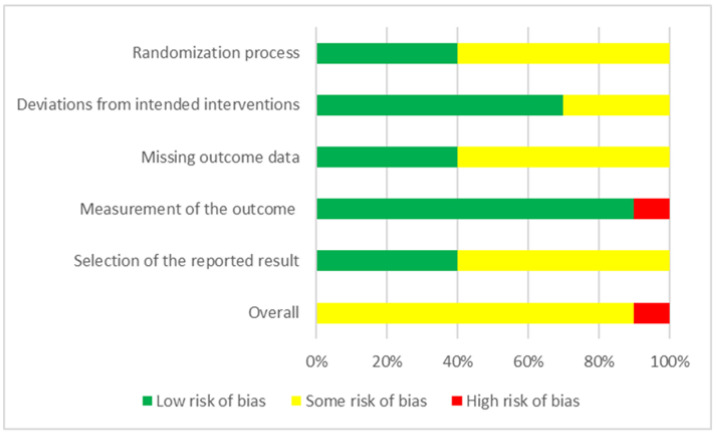
Summary of quality assessment of studies included in the meta-analysis using version 2 of the Cochrane Risk-of-Bias Tool for Randomized Trials.

During the final stage of selecting studies for systematic review, a quality assessment was conducted. Two authors (C.-H. Chang and W.-J. Chen) carried out this quality assessment, and in cases of disagreement, consensus was reached through discussion. To evaluate the methodological quality of the studies included in our analysis, we used the Cochrane Risk-of-Bias Tool for Randomized Trials Version 2 (RoB 2), which comprises 6 main criteria, namely, randomization process, intervention adherence, missing outcome data, outcome measurement, selective reporting, and overall risk of bias [34]. For the intervention adherence section of RoB 2, an assessment of the literature required the selection of 1 of 2 options: intention-to-treat (intervention assignment) or per-protocol (intervention adherence). For our meta-analysis, we selected the per-protocol evaluation approach [39] (Figure 2).

### 2.5. Primary Outcome (Changes in Depression Scores)

The primary outcome was changes in depression scores following either NIBS or placebo treatment. The validity and appropriacy of the depression scale used in each trial were examined by investigating pertinent references, and the depression assessment scales used in each study were examined. If 2 scales were identified for assessing depression, the main depression assessment scale used in the study in question or the scale with pretest and post-test results was selected. If the depression outcomes were measured at multiple time points—such as immediately after treatment, 1 month after treatment, and 2 months after treatment—the immediate assessment result was prioritized. If a study had no immediate assessment result, the most recent assessment result, such as the assessment result obtained 1 month after treatment, was selected.

### 2.6. Secondary Outcomes (Adverse Effect Rates, Seizures, and Suicide)

The secondary outcomes were adverse event rates, seizures, and suicide, and these outcomes were quantified using odds ratios (ORs). According to these ORs, in the NIBS group, adverse event rates were the most prevalent secondary outcome [40].

### 2.7. Data Integration and Statistical Evaluation

In this paper, the results of depression scores are presented as SMDs. A positive SMD value indicated a decrease in depressive symptoms after either NIBS or sham treatment. SMD for each study was calculated by the reported mean and standard deviation. For those studies not providing standard deviation [25,27,37], we transformed and obtained it by other provided statistics (i.e., t value, *p* value). Of the ten included studies, none exhibited missing data or reported dropouts. A random-effects model was employed to collect SMD data from multiple sources. A 2-tailed statistical analysis was conducted at a significance level of 0.05. Cohen’s *d* and 95% CIs were used to quantify the primary outcome (changes in depression scores). In addition, *I*^2^ statistics were used to determine the degree of variation between trials; a value above 50% was used to indicate substantial heterogeneity. We used subgroup analysis and meta-regression to analyze the moderators including the NIBS type, frequency, brain target, TBI baseline severity, total number of sessions, total pulses, and treatment intensity (defined as % Resting Motor Threshold (RMT) referring to the minimum amount of magnetic field strength required to elicit a motor-evoked potential in a target muscle). Furthermore, a sensitivity analysis in which a single study was eliminated at a time was conducted to evaluate the effect of the eliminated study on the remaining studies. To determine the likelihood of publication bias, both funnel plots and Egger’s test were used. Meta-analysis and meta-regression were conducted using Comprehensive Meta-Analysis software version 3.0 (Biostat, Englewood, NJ, USA).

## 3. Results

### 3.1. Characteristics of the Included Studies

A total of 10 studies [22,23,24,25,26,27,33,36,37,38], involving a total of 234 patients with TBI (mean age: 41.07 ± 7.26 years, 63.74% men), were included in the final analysis. The average number of participants in each study was 23.40 ± 6.87 (range: 13–32), and the average number of treatment sessions was 10.60 ± 6.81 (range: 1–20). The mean time since injury was 9.79 ± 5.29 years, and the mean baseline depression score was 18.49 ± 10.17. The following 5 depression measure scales were used: Patient Health Questionnaire-9 (2 studies, *n* = 46) [41], the Profile of Mood States (POMS; 1 study, *n* = 30) [42], the Montgomery–Åsberg Depression Rating Scale (MADRS; 4 studies, *n* = 67), the Hamilton Rating Scale for Depression (HAMD; 2 studies, *n* = 59) [43], and the Beck Depression Inventory (1 study, *n* = 32) [44]. HAMD and MADRS are questionnaires used by psychiatrists and researchers to measure the severity of depression. The BDI is a 21-item, self-report rating inventory that measures characteristic attitudes and symptoms of depression. HAMD, MADRS, and BDI are commonly used in depression research. The PHQ-9 is a diagnostic tool used to screen adult patients in primary care settings for the presence and severity of depression. POMS is a psychological rating scale employed to assess transient, distinct mood states. Among the 10 analyzed studies, 8 involved a single scale for the evaluation of depression, and 2 involved more than one scale. For instance, Rushby et al. [33] used both the POMS and HAMD. For that study, we selected the POMS over the HAMD because the HAMD yielded no post-test data. Hoy et al. [26] used 3 scales—namely, the MADRS, the Inventory for Depressive Symptomatology—Clinician-Rated Version, and the Inventory for Depressive Symptomatology—Self-Rated Version—with the MADRS being the primary depression assessment scale. Table 1 summarizes the characteristics of the included studies. By using RoB 2 to evaluate study quality, we discovered that 90% and 10% of the studies had a certain risk of bias and a high risk of bias, respectively (Figure 2).

### 3.2. Meta-Analysis Results of the Overall Effects of NIBS

**Figure 3:**
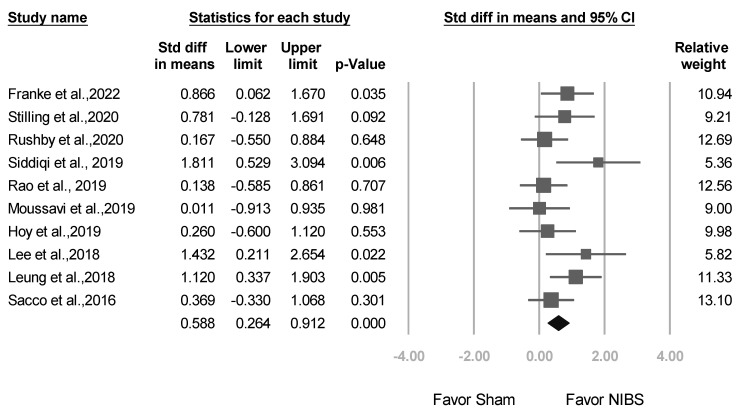
Meta-analysis results of all studies in terms of SMDs in depression scores. (Note: in the graph, the square represents the effect size of each study. The bigger the square, the more participants in the study. A horizontal line represents the 95% confidence intervals of the study result, with each end of the line representing the boundaries of the confidence interval. The diamond represents the combined effect). Franke et al., 2022 [25]; Stilling et al., 2020 [36]; Rushby et al., 2020 [33]; Siddiqi et al., 2019 [22]; Rao et al., 2019 [37]; Moussavi et al., 2019 [38]; Hoy et al., 2019 [26]; Lee et al., 2018 [23]; Leung et al., 2018 [24]; Sacco et al., 2016 [27].

Positive SMD results were obtained indicating the alleviation of clinical symptoms after adjunct NIBS. Compared with sham treatment, NIBS exhibited higher efficacy in reducing depression scores (SMD: 0.588, 95% CI: 0.264–0.912; *p* < 0.001; Figure 3).

### 3.3. Meta-Analysis Results of Studies Stratified by Different Factors

#### 3.3.1. Studies Stratified by NIBS Type

**Figure 4:**
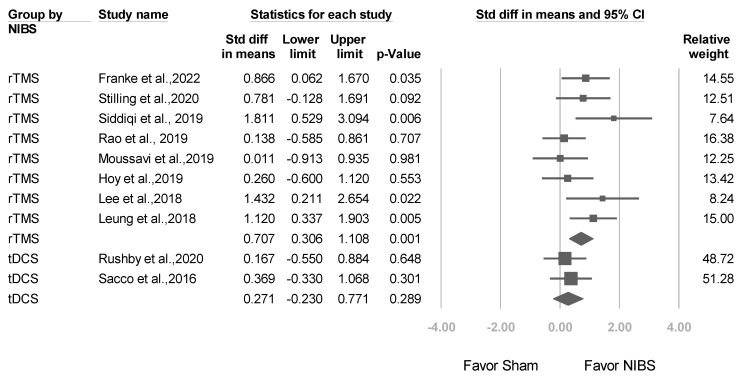
Meta-analysis results of studies stratified by NIBS type in terms of SMDs in depression scores. (Note: in the graph, the square represents the effect size of each study. The bigger the square, the more participants in the study. A horizontal line represents the 95% confidence intervals of the study result, with each end of the line representing the boundaries of the confidence interval. The diamond represents the combined effect). Franke et al., 2022 [25]; Stilling et al., 2020 [36]; Siddiqi et al., 2019 [22]; Rao et al., 2019 [37]; Moussavi et al., 2019 [38]; Hoy et al., 2019 [26]; Lee et al., 2018 [23]; Leung et al., 2018 [24]; Rushby et al., 2020 [33]; Sacco et al., 2016 [27].

A total of 8 trials used rTMS, whereas tDCS was used in 2 trials. As shown in Figure 4, subgroup meta-analysis revealed that compared with sham treatment, rTMS had significantly higher efficacy for reducing depression scores (SMD: 0.707, 95% CI: 0.306–1.108; *p* = 0.001). However, nonsignificant corresponding results were obtained for tDCS (SMD: 0.271, 95% CI: −0.230 to 0.771; *p* = 0.289; *p* value for interaction = 0.182).

#### 3.3.2. Studies Stratified by Stimulation Frequency

**Figure 5:**
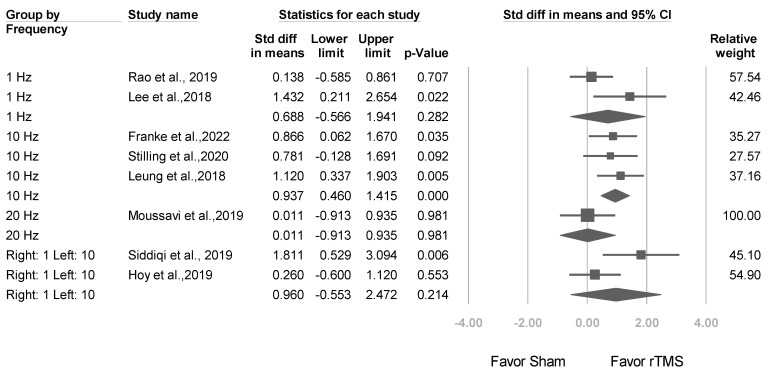
Meta-analysis results of studies stratified by stimulation frequency in terms of SMDs in depression scores. (Note: in the graph, the square represents the effect size of each study. The bigger the square, the more participants in the study. A horizontal line represents the 95% confidence intervals of the study result, with each end of the line representing the boundaries of the confidence interval. The diamond represents the combined effect). Rao et al., 2019 [37]; Lee et al., 2018 [23], Franke et al., 2022 [25]; Stilling et al., 2020 [36]; Leung et al., 2018 [24]; Moussavi et al., 2019 [38]; Siddiqi et al., 2019 [22]; Hoy et al., 2019 [26].

In 3 trials involving a stimulation frequency of 10 Hz, significant effect sizes were reported (mean effect size: 0.937, 95% CI: 0.460–1.415; *p* < 0.001; Figure 5). By contrast, in 2 trials involving a stimulation frequency of 1 Hz, nonsignificant effect sizes were reported (mean effect size: 0.688, 95% CI: −0.566 to 1.941; *p* = 0.282).

#### 3.3.3. Studies Stratified by Brain Target

**Figure 6:**
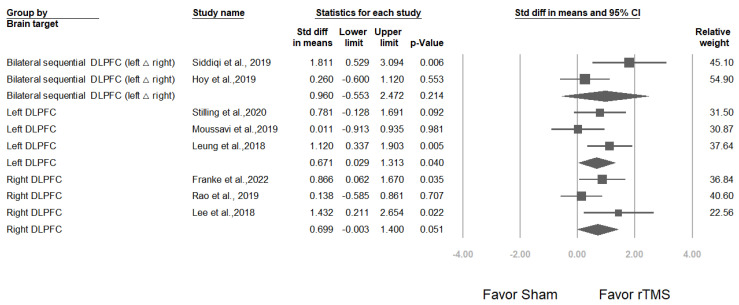
Meta-analysis results of studies stratified by brain target, in terms of SMDs in depression scores. (Note: in the graph, the square represents the effect size of each study. The bigger the square, the more participants in the study. A horizontal line represents the 95% confidence intervals of the study result, with each end of the line representing the boundaries of the confidence interval. The diamond represents the combined effect). Siddiqi et al., 2019 [22]; Hoy et al., 2019 [26]; Stilling et al., 2020 [36]; Moussavi et al., 2019 [38]; Leung et al., 2018 [24]; Franke et al., 2022 [25]; Rao et al., 2019 [37]; Lee et al., 2018 [23].

Two studies [22,26] targeted the bilateral sequential dorsolateral prefrontal cortex (DLPFC), with an overall effect size of 0.960 (95% CI: −0.533 to 2.472; *p* = 0.214); 3 studies [24,36,38] targeted the left DLPFC, with an overall effect size of 0.671 (95% CI: 0.029–1.313; *p* = 0.040), and 3 studies [23,25,37] targeted the right DLPFC, with an overall effect size of 0.699 (95% CI: −0.003 to 1.400; *p* = 0.051) (Figure 6). Mixed-effects analysis was conducted to evaluate the total difference between modulators for these 3 different brain targets, revealing a nonsignificant difference (*p* value for interaction = 0.942).

#### 3.3.4. Studies Stratified by Baseline TBI Severity

**Figure 7:**
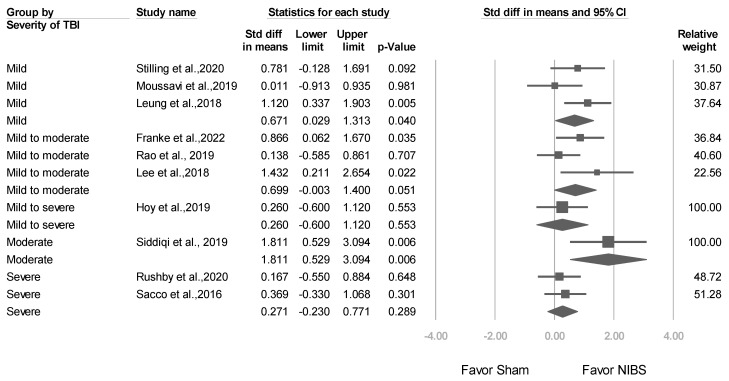
Meta-analysis results of studies stratified by baseline TBI severity in terms of SMDs in depression scores. (Note: in the graph, the square represents the effect size of each study. The bigger the square, the more participants in the study. A horizontal line represents the 95% confidence intervals of the study result, with each end of the line representing the boundaries of the confidence interval. The diamond represents the combined effect). Stilling et al., 2020 [36]; Moussavi et al., 2019 [38]; Leung et al., 2018 [24]; Franke et al., 2022 [25]; Rao et al., 2019 [37]; Lee et al., 2018 [23]; Hoy et al., 2019 [26]; Siddiqi et al., 2019 [22]; Rushby et al., 2020 [33]; Sacco et al., 2016 [27].

Three trials [24,36,38] included patients with mild TBI (mean effect size: 0.671, 95% CI: 0.029–1.313; *p* = 0.040), 3 trials [23,25,37] included patients with mild-to-moderate TBI (mean effect size: 0.699, 95% CI: −0.003 to 1.400; *p* = 0.051), and 2 trials [27,33] included patients with severe TBI (mean effect size: 0.271, 95% CI: −0.230 to 0.771; *p* = 0.289) (Figure 7). Mixed-effects analysis revealed a nonsignificant difference (*p* value for interaction = 0.227).

### 3.4. Meta-Regression Analysis Results

**Figure 8:**
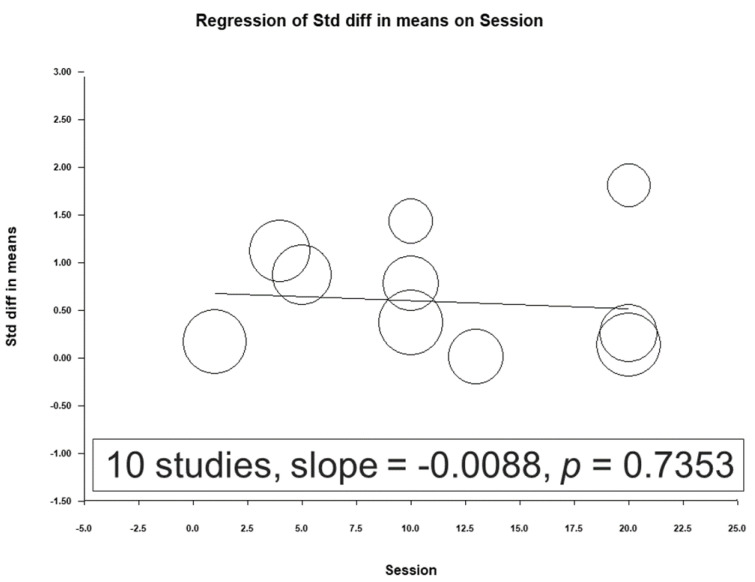
Meta-regression results of the association between the efficacy of rTMS and the total number of sessions.

**Figure 9:**
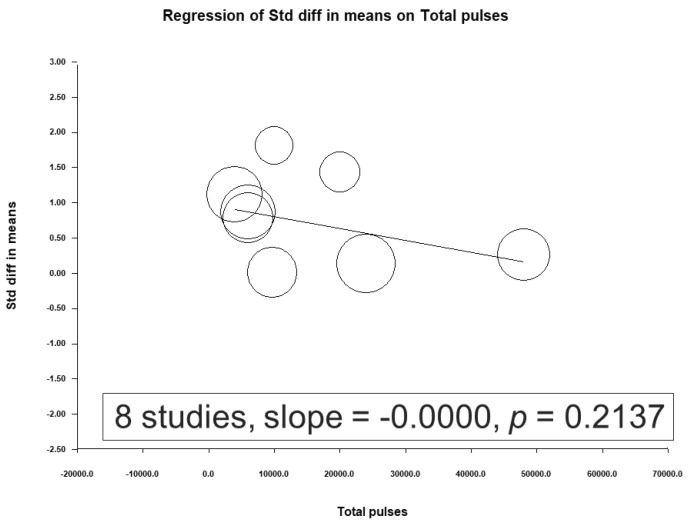
Meta-regression results of the association between the efficacy of rTMS and the total pulses.

**Figure 10:**
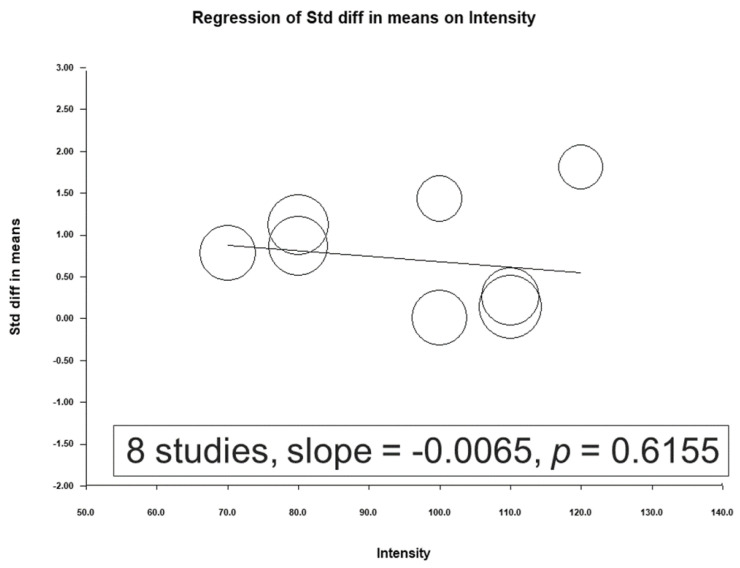
Meta-regression results of the association between the efficacy of rTMS and treatment intensity.

A meta-regression was conducted using the total number of sessions, total pulses, and treatment intensity as moderators (Figure 8, Figure 9 and Figure 10). No significant association was observed between the effect sizes and these moderators.

### 3.5. Short- and Long-Term Effects after NIBS Treatment

**Figure 11:**
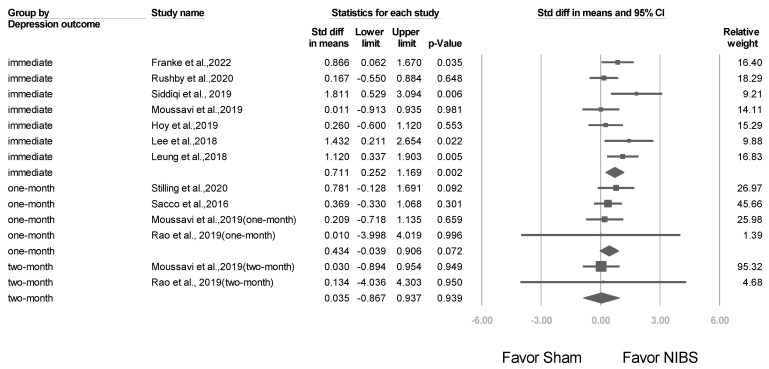
Forest plot indicating the efficacy of NIBS versus sham treatment for the treatment of depression. The depression outcome was evaluated immediately, 1 month, and 2 months after NIBS treatment. (Note: in the graph, the square represents the effect size of each study. The bigger the square, the more participants in the study. A horizontal line represents the 95% confidence intervals of the study result, with each end of the line representing the boundaries of the confidence interval. The diamond represents the combined effect). Franke et al., 2022 [25]; Rushby et al., 2020 [33]; Siddiqi et al., 2019 [22]; Moussavi et al., 2019 [38]; Hoy et al., 2019 [26]; Lee et al., 2018 [23]; Leung et al., 2018 [24]; Stilling et al., 2020 [36]; Sacco et al., 2016 [27]; Rao et al., 2019 [37].

A total of 7 studies were included in this meta-analysis to evaluate the effect of treatment on the outcome of interest. The results obtained immediately after treatment demonstrated an overall effect size of 0.711 (95% CI: 0.252–1.169; *p* = 0.002). Four studies reported results 1 month after treatment, with an overall effect size of 0.434 (95% CI: −0.039 to 0.906; *p* = 0.072), whereas 2 studies reported results 2 months after treatment, with an overall effect size of 0.035 (95% CI: −0.867 to 0.937; *p* = 0.939). Figure 11 graphically summarizes these findings.

### 3.6. Secondary Outcomes: Adverse Event Rates, Seizures, and Suicide

**Figure 12:**
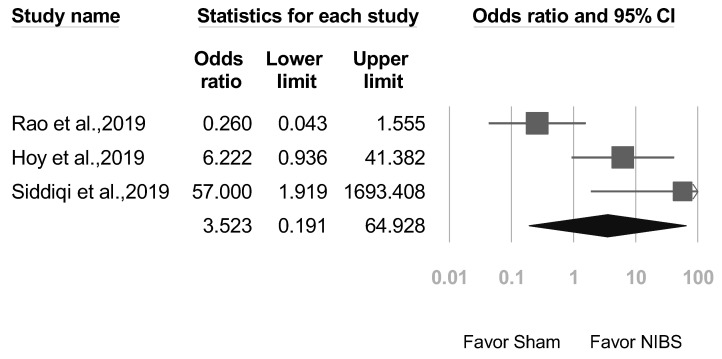
Forest plot of adverse event rates for NIBS treatment versus sham treatment. (Note: in the graph, the square represents the effect size of each study. The bigger the square, the more participants in the study. A horizontal line represents the 95% confidence intervals of the study result, with each end of the line representing the boundaries of the confidence interval. The diamond represents the combined effect). Rao et al., 2019 [37].; Hoy et al., 2019 [26]; Siddiqi et al., 2019 [22].

No serious adverse effects such as seizures were reported in any of the included studies. The common side effects of NIBS were headache, transient twitching, and facial muscle discomfort. A total of 3 studies reported adverse effects. As shown in Figure 12, a summary meta-analysis revealed no statistical significance; all recorded adverse events were minor ones. In addition, no suicidal incidents were reported during or after NIBS treatment. A total of 2 studies reported the alleviation of suicidal ideation following NIBS treatment. Rao et al. [37] used the Beck Scale for Suicide Ideation to examine attitudes toward suicide (Hedges’ *g* effect size = 0.21). Siddiqi et al. [22] reported improvements in MADRS subscores for suicidal thoughts after both active treatment and sham treatment (Cohen’s *d* = 1.75).

### 3.7. Heterogeneity and Publication Bias

**Figure 13:**
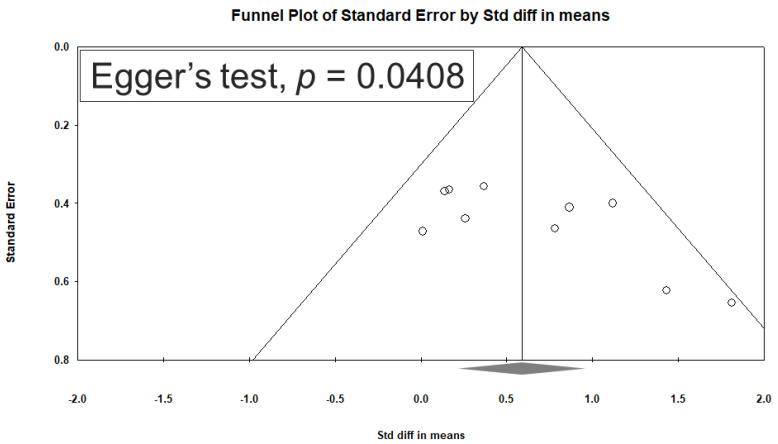
Funnel plot constructed for SMDs among patients’ depression scores.

No significant heterogeneity was observed across the 10 included studies (*Q* = 12.932, df = 9, *I*^2^ = 30.408%; *p* = 0.166). Egger’s test revealed significant publication bias in terms of the overall SMD (*p* = 0.0408). Figure 13 depicts a funnel plot constructed for SMDs among patients’ depression scores.

### 3.8. Sensitivity Analysis Results

Even after individual studies were eliminated, our results regarding the efficacy of NIBS remained significant.

## 4. Discussion

To the best of our knowledge, the present study was the first meta-analysis to examine the efficacy of NIBS for treating patients with TBI. We discovered that NIBS is effective for alleviating depressive symptoms in patients with post-TBI depression, regardless of the NIBS type, brain target, number of sessions, treatment intensity, or total dose. We identified no severe side effects, such as seizures or suicide, across the included studies.

### 4.1. Comparison with Previous Meta-Analyses

Our findings are consistent with those of a meta-analysis conducted by Tsai et al. [29]. In our review, which included 10 studies (8 on rTMS and 2 on tDCS), we discovered that compared with sham treatment, NIBS treatment exhibited significantly higher efficacy in alleviating depressive symptoms (SMD: 0.588, 95% CI: 0.264–0.912; *p* < 0.001). Tsai et al. [29] reviewed 7 studies on rTMS and discovered that rTMS was effective against depression (SMD: 1.03, 95% CI: 0.20–1.86; *p* = 0.02). In the present study, we also discovered that rTMS treatment was more effective than sham treatment in reducing depression scores. Specifically, our subgroup meta-analysis revealed that compared with sham treatment, rTMS treatment exhibited significantly higher efficacy in reducing depression scores (SMD: 0.707, 95% CI: 0.306–1.108; *p* = 0.001). By contrast, the results obtained for tDCS were nonsignificant (SMD: 0.271, 95% CI: −0.230 to 0.771; *p* = 0.289). According to these findings, the differences in efficacy between rTMS and tDCS may correlate with the number of analyzed trials and the mechanisms underlying the effects of the 2 techniques. In addition, the relatively large number of rTMS trials may have contributed to the significance of the corresponding results. Furthermore, the mechanisms of action (for depression treatment) may vary between rTMS and tDCS. In rTMS, a magnetic field is indirectly created across the skull, generating an electric current in the targeted brain regions [10,11]. By contrast, in tDCS, an electric current (typically 1–2 mA) is directly delivered through 2 or more electrodes placed on the patient’s scalp [17]; the resultant current penetrates the skull and modulates the neural activity of the targeted brain regions [18]. In a meta-analytical study of 49 trials involving 2941 patients with treatment-resistant depression, rTMS was determined to be more effective than tDCS. Compared with sham treatment, multiple forms of transcranial magnetic stimulation—including bilateral theta burst stimulation (TBS; relative risk: 5.00, 95% CI: 1.11–22.44), low-frequency right rTMS (relative risk: 2.62, 95% CI: 1.56–4.39), high-frequency left rTMS (relative risk: 2.18, 95% CI: 1.52–3.13), bilateral rTMS (relative risk: 3.08, 95% CI: 1.78–5.31), and tDCS (relative risk: 0.85, 95% CI: 0.05–13.09)—exhibited higher response rates [45]. Another meta-analytical study regarding post-stroke depression revealed that the rTMS group experienced greater alleviation of depressive symptoms compared with the sham rTMS group, with an SMD of 4.92 (95% CI: 2.69–7.15, *I*^2^ = 95.2%; *p* < 0.001), and that the tDCS group experienced greater alleviation of depressive symptoms compared with the sham tDCS group, with an SMD of 5.30 (95% CI: 1.30–9.30, *I*^2^ = 97.3%; *p* < 0.001) [46]. Nevertheless, further studies are required to determine the effects of various brain stimulation techniques on post-TBI depression.

### 4.2. High-Frequency vs. Low-Frequency rTMS Efficacy

Among the 8 rTMS studies included in the present meta-analysis [22,23,24,25,26,36,37,38], 7 reported major alleviation of depressive symptoms [16,17,18,19,20,22,23], and 1 reported minor alleviation (Hedges’ *g* = 0.19) for TBI-related depression [37]. Rao et al. [37] targeted the right DLPFC with low-frequency rTMS; however, in the present study, we discovered that high-frequency (10 Hz) rTMS was more effective than low-frequency (1 Hz) rTMS for alleviating depressive symptoms. Our findings are consistent with those of other meta-analyses regarding major depression and post-stroke depression. In a systematic review and meta-analysis of studies in which rTMS was used to treat patients who experienced major depressive episodes, Brunoni et al. [47] reported treatment responses of 3.07 (2.24–4.21) and 2.37 (1.52–3.68) after high- and low-frequency rTMS, respectively. In another meta-analysis of studies evaluating the effects of rTMS on post-stroke depression, Shen et al. [48] reported that high-frequency (≥10 Hz; *n* = 10) rTMS exerted stronger effects than did low-frequency (≤1 Hz) rTMS, with mean differences of −6.20 [−9.21, −3.19] and −5.40 [−7.56, −3.23], respectively. Notably, the aforementioned studies have focused on depression rather than TBI, and thus further studies are warranted to compare the effects of high- and low-frequency rTMS on patients with TBI. NIBS techniques, rTMS and tDCS, function distinctively. rTMS induces electrical currents in the brain via magnetic pulses, with high-frequency stimulation typically enhancing cortical excitability and low-frequency dampening it. tDCS, on the other hand, modulates neuronal membrane potentials—either depolarizing (anodal) or hyperpolarizing (cathodal) neurons. For post-TBI depression, high-frequency rTMS could target hypoactive regions like the left DLPFC, elevating its activity, while low-frequency might suppress overactive areas. tDCS could be optimized by tailoring polarity based on desired excitability shifts. Combining these protocols or alternating them might offer synergistic effects, capitalizing on their complementary mechanisms for holistic treatment.

### 4.3. Brain Targets in NIBS Treatment Studies

The present study evaluated the efficacy of NIBS in relation to multiple brain targets and discovered that the left DLPFC was targeted in 3 studies [24,36,38], the right DLPFC was targeted in 3 studies [23,25,37], and the bilateral DLPFC was targeted in 2 studies [17,22]. Targeting the left DLPFC was associated with significantly more favorable outcomes (SMD: 0.671; *p* = 0.004) compared with targeting the right DLPFC or bilateral DLPFC (SMDs: 0.699 and 0.960, respectively; *p* = 0.051 and 0.214, respectively). These findings corroborate those of Tsai et al. [29], who also reported that targeting the left DLPFC was associated with more favorable outcomes (SMD: 0.98; *p* = 0.04) compared with targeting the bilateral DLPFC or right DLPFC (SMD: 1.44 and 0.99, respectively; *p* = 0.25 and 0.44, respectively). The DLPFC is pivotal in cognitive function and emotional regulation. In depression, reduced activity and connectivity in the DLPFC relate to the symptomatology, whereas TBI can disrupt its structural and functional integrity, exacerbating depressive symptoms. Targeting the DLPFC with treatments like NIBS can potentially restore its function, thereby ameliorating depressive symptoms. While unilateral stimulation (typically left DLPFC) has been favored for its antidepressant effects, bilateral stimulation—combining excitatory stimulation of the left DLPFC with inhibitory stimulation of the right—may offer enhanced efficacy by simultaneously elevating activity in one hemisphere and dampening potential overactivity in the other.

### 4.4. Factors Influencing Clinical Outcomes in TBI Patients

The correlations of NIBS efficacy with multiple influencing factors were further evaluated in this study. A meta-regression was conducted with moderators, including the total number of sessions, total pulses, and treatment intensity. No significant association was observed between the effect sizes and these moderators. In a systematic review of factors influencing the clinical outcomes of patients with TBI, Kim [49] reported that the following factors were associated with poor clinical outcomes: advanced age, male sex, low educational level, low Glasgow Coma Scale score, injury caused by a motor vehicle crash, hypotension, hypoxia, high intracranial pressure, no pupil reaction, hypoglycemia or hyperglycemia, anemia, coagulopathy, hypothermia or hyperthermia, abnormal electrolyte levels, coma duration, high Marshall classification (computed tomography) level, and intracerebral lesion type. Further studies are required to determine the effects of various factors on the outcomes of TBI-related depression.

### 4.5. Possible Pathophysiological Mechanisms

Studies regarding depression have extensively investigated the effects of rTMS on the DLPFC [47]. However, despite the widespread use of rTMS, the specific mechanism underlying its treatment response remains not well understood. Some researchers have suggested that high-frequency rTMS may affect the brain architecture, resulting in an increased gray matter volume after treatment [50]. Other researchers have proposed that rTMS may induce changes in cerebral hemodynamic response and functional connectivity, both of which play a crucial role in patients with persistent post-traumatic headaches and post-concussive syndrome [51,52,53,54]. NIBS techniques, such as rTMS and tDCS, appear to alleviate post-TBI depression by modulating neural plasticity, thereby aiding disrupted neural pathways common after TBI. They also adjust cortical excitability, potentially addressing the hypoactivity seen in depression-related regions like the DLPFC. Furthermore, NIBS may normalize neurotransmitter imbalances, a shared pathology in both TBI and depression. The neuroinflammatory response, heightened post-TBI, and link to depression onset, might be mitigated by NIBS’s possible anti-inflammatory effects. In essence, the therapeutic potential of NIBS in post-TBI depression likely stems from its capacity to address overlapping neuropathological changes inherent in both TBI and depression. Further studies may benefit from the incorporation of additional modalities, such as functional magnetic resonance imaging or functional near-infrared spectroscopy, to better understand the physiological mechanisms of brain stimulation [55].

### 4.6. Safety and Tolerability of NIBS for TBI Patients

The analysis of secondary outcomes in the provided studies underscores the promising safety profile of NIBS, particularly for TBI patients. While there were instances of common side effects, such as headaches, transient twitching, and facial muscle discomfort, the absence of serious adverse events like seizures is encouraging. As detailed in Figure 12, a comprehensive meta-analysis revealed that all adverse events documented were mild in nature. Notably, the procedure seems to have had a beneficial effect on patients’ mental health, as evidenced by the absence of reported suicidal incidents during or post treatment and the noted alleviation of suicidal ideation in certain studies. To further optimize the safety and tolerability of NIBS for TBI patients, certain precautions are recommended. Clinicians should conduct a meticulous pre-treatment evaluation to detect any potential risks and ensure patient suitability. Precise electrode placement, adhering to recommended current levels, and continuous monitoring during the procedure can also mitigate the emergence of side effects. Such careful measures reaffirm the commitment to providing TBI patients with a safe and effective therapeutic intervention.

### 4.7. Strengths

This study had several strengths. First, we included 10 trials, whereas previous related studies [28,29,30] have included fewer than 10 trials. Second, in addition to rTMS trials, we included 2 tDCS trials. Third, we evaluated the correlations of NIBS efficacy with several modulators, including the total number of sessions, total pulses, and treatment intensity. Fourth, we evaluated factors such as adverse effects, seizures, and suicide.

### 4.8. Limitations

Notably, this study also had some limitations. First, the numbers of included trials and analyzed patients were small. No significant effect was found at 1 month or 2 months. Second, brain lesions involving or not involving the DLPFC and other comorbidities affected NIBS treatment. Among the 10 trials reviewed in this meta-analysis, 8 did not report the specific locations of brain injury in the participants, and the remaining 2 excluded patients with frontal lobe injuries. Furthermore, of these 10 trials, only 3 reported comorbidities. Therefore, further studies are required to determine the effects of frontal lobe involvement (especially that including the DLPFC) and comorbidities on the efficacy of brain stimulation therapy. Third, the protocols used to evaluate the efficacy of NIBS for the treatment of depression varied across the included trials. Three studies [27,33,38] included were not designed to assess efficiency on depressive symptoms as a primary outcome. Fourth, our sample did not include non-randomized trials or unpublished studies. Fifth, we did not evaluate concomitant medication nor genetic or psychosocial factors that may serve as potential confounders. The inclusion of all type of TBI from mild to severe in the same meta-analysis need further evaluation because the physiopathology is dramatically different between the two conditions. Sixth, we did not evaluate advanced rTMS protocols such as TBS, which involves the delivery of short high-frequency (50 Hz) pulses (5 Hz at intervals of 200 ms, intermittent or continuous TBS) to rapidly induce synaptic plasticity [56]. Seventh, the scales used for assessing depressive symptoms were not able to discriminate depressive symptoms from the neuropsychiatric symptoms directly related to TBI such as apathy, poor concentration, or fatigue. Eighth, small sample sizes, heterogeneity in protocols, lack of control groups, short follow-up periods, lack of replication, and potential confounds were not addressed. Accordingly, further high-quality RCT studies with larger sample sizes are warranted to investigate these limitations.

## 5. Conclusions

This meta-analysis underscores rTMS as an effective short-term treatment for depression in TBI patients. However, its benefits diminish within 1–2 months post intervention, indicating the need for sustained or supplementary therapies. The inconclusive results for tDCS require further investigation, while the overall low adverse event rate supports NIBS’s safety profile. Future research should prioritize long-term efficacy and strategies for maintaining therapeutic gains.

## Figures and Tables

**Table 1:** Characteristics of studies included in our meta-analysis.

Study (First Author, Year)	Patient Population	N	Gender (%Male)	Mean Age (Years)	Depression Measure	Baseline Mean Depression Scores	Depression Outcome	NIBS	Brain Target	Frequency	Intensity (% RMT)	Sessions	Total Dose (Pulses)	Time Since Injury
Franke et al., 2022 [25]	Mild-to-moderate TBI	26	85.7	45.57 (10.01)	PHQ-9	10.07 (5.33)	immediate two-week	rTMS	Right DLPFC	10 Hz	80	5	6000	12.04 (6.80) years
Stilling et al., 2020 [36]	Mild TBI	20	10.0	36.0 (11.4)	PHQ-9	11.90 (6.74)	one-month	rTMS	Left DLPFC	10 Hz	70	10	6000	32.5 (13.9) months
Rushby et al., 2020 [33]	Severe TBI	30	70.0	50.0 (1.1)	POMS Depression HAMD	2.2 (2.8)	immediate	tDCS	left inferior parietal cortex (corresponding to the P3 location) and the cathode was placed over the right inferior parietal cortex (P4 location)	2 mA of tDCS for 20 min	Not applicable	1	NA	13.9 (12.1) years
Siddiqi et al., 2019 [22]	Moderate TBI	15	73.3	45.8 (15.1)	MADRS	32.2	immediate	rTMS	Bilateral sequential DLPFC (left → right)	Right: 1 Hz Left: 10 Hz	120	20	10,000	8.3 (9.5) years
Rao et al., 2019 [37]	Mild to moderate TBI	30	53.3	40.0 (14.4)	HAMD	23.5 (4.4)	immediate one-month two-month three-month	rTMS	Right DLPFC	1 Hz	110	20	24,000	3 months to >10 years
Moussavi et al., 2019 [38]	Mild TBI	18	50.0	49.9 (12.5)	MADRS	14.6 (8.8)	immediate one-month two-month	rTMS	Left DLPFC	20 Hz	100	13	9750	1.7 (1.3) years
Hoy et al., 2019 [26]	Mild to severe TBI	21	47.6	46.3 (11.7)	MADRS IDS-CR IDS-SR	34.0 (8.0)	immediate	rTMS	Bilateral sequential DLPFC (right → left)	Right: 1 Hz Left: 10 Hz	110	20	48,000	18.2 (12.2) years
Lee et al., 2018 [23]	Mild to moderate TBI	13	69.2	42.0 (11.2)	MADRS	23.8 (4.3)	immediate	rTMS	Right DLPFC	1 Hz	100	10	20,000	NA
Leung et al., 2018 [24]	Mild TBI	29	79.3	34.1 (7.9)	HAMD	23.9 (7.5)	immediate	rTMS	Left DLPFC	10 Hz	80	4	4000	97.1 (71.1) months
Sacco et al., 2016 [27]	Severe TBI	32	50.0	18 to 66	BDI	NA	one month	tDCS	F3 or F4 anodal (anode on the lesioned hemisphere and cathode on the other hemisphere); bi-montage F3/F4 anodal in case of equal hemispheric lesion distribution	10 min of anodal tDCS, 1 mA	Not applicable	10	NA	3.16 to 17.5

Abbreviations: BDI, Beck Depression Inventory; DLPFC, dorsolateral prefrontal cortex; HAMD, Hamilton Rating Scale for Depression; MADRS, Montgomery–Åsberg Depression Rating Scale; NA, not available; PHQ-9, Patient Health Questionnaire-9; POMS, Profile of Mood States; rTMS, repetitive transcranial magnetic stimulation; TBI, traumatic brain injury; tDCS, transcranial direct current stimulation; IDS-CR, Inventory for Depressive Symptomatology—Clinician-Rated Version; IDS-SR, Inventory for Depressive Symptomatology—Self-Rated Version.

## Data Availability

Details of all data generated or analyzed in this study are included in this published manuscript.

## Supplementary Materials

The following supporting information can be downloaded at: https://www.mdpi.com/article/10.3390/jcm12186030/s1, Table S1: Search Terms and Strings Utilized for Each Database.

## List of Abbreviations

BDI: Beck Depression Inventory; DLPFC: dorsolateral prefrontal cortex; HAMD: Hamilton Rating Scale for Depression; rTMS: repetitive transcranial magnetic stimulation; TBS: theta burst stimulation.
