# Supplementary material for: Efficacy of Non-Invasive Brain Stimulation for Treating Depression in Patients with Traumatic Brain Injury: A Meta-Analysis and Meta-Regression of Randomized Controlled Trials
Source: J Clin Med. 2023 Sep 18;12(18):6030. doi: 10.3390/jcm12186030 (PMC10531948; doi:10.3390/jcm12186030)
Supplement: Supplementary file 1 [file jcm-12-06030-s001.zip › jcm-2415208-supplementary.pdf]

Table S1: Search Terms and Strings Utilized for Each Database

Database	Keywords:
PubMed	(traumatic brain injur* OR TBI OR head injur* OR brain injur* OR brain trauma OR concussion OR concussive) AND (tDCS OR transcranial direct current stimulation OR non-invasive stimulation OR transcranial magnetic stimulation OR TMS OR rTMS OR brain stimulation) AND controlled trial
Cochrane Central Register of Controlled Trials	(traumatic brain injur* OR TBI OR head injur* OR brain injur* OR brain trauma OR concussion OR concussive) AND (tDCS OR transcranial direct current stimulation OR non-invasive stimulation OR transcranial magnetic stimulation OR TMS OR rTMS OR brain stimulation)
and Cochrane Database of Systematic Reviews	(traumatic brain injur* OR TBI OR head injur* OR brain injur* OR brain trauma OR concussion OR concussive) AND (tDCS OR transcranial direct current stimulation OR non-invasive stimulation OR transcranial magnetic stimulation OR TMS OR rTMS OR brain stimulation)
